# eIf3a mediates malignant biological behaviors in colorectal cancer through the PI3K/AKT signaling pathway

**DOI:** 10.1080/15384047.2024.2355703

**Published:** 2024-05-23

**Authors:** Chao Huo, Disheng Wu, Xiaodan Li, Yan Zhang, Baoguang Hu, Taoming Zhang, Jianwei Ren, Tianbao Wang, Yi Liu

**Affiliations:** a Department of Anus and Intestines, Shenzhen Nanshan People’s Hospital, Shenzhen, Guangdong, China; b Hongshan Community Hospital, People’s Hospital of Longhua District, Shenzhen, Guangdong, China; c Guangdong Provincial Key Laboratory of Research and Development of Natural Drugs, School of Pharmacy, Guangdong Medical University, Dongguan, Guangdong, China; d Department of Gastrointestinal Surgery, Binzhou Medical University Hospital, Binzhou, Shandong, China; e Centre for Regenerative Medicine and Health, Hong Kong Institute of Science and Innovation, Chinese Academy of Sciences, Hong Kong, China; f R&D Department, Shenzhen Ritzcon Biological Technology Co., Ltd., Shenzhen, Guangdong, China; g Department of Gastrointestinal Surgery, South China Hospital of Shenzhen University, Shenzhen, Guangdong, China; h School of Ocean and Tropical Medicine, Guangdong Medical University, Zhanjiang, Guangdong, China

**Keywords:** Colorectal cancer, e3IFa, PI3K/AKT, tumor growth, malignant behaviors

## Abstract

Colorectal cancer (CRC) is among the most common gastrointestinal malignancies worldwide. eIF3a is highly expressed in a variety of cancer types, yet its role in CRC remains unclear. We introduced ectopic eIF3a expression in CRC cells to investigate its relevance to various malignant behaviors. Further, we silenced eIF3a to explore its effect on tumor growth in a nude mouse tumor xenograft model. Finally, the molecular mechanisms through which eIF3a regulates malignancy in CRC cells were explored through bioinformatics analysis combined with the use of a specific PI3K inhibitor (LY294002). eIF3a was highly expressed in the peripheral blood and cancer tissue of CRC patients. Malignancy and tumor growth were significantly inhibited by silencing eIF3a, while overexpression promoted malignant behaviors, with a positive correlation between PI3K/AKT activation and eIF3a expression. Taken together, eIF3a plays an oncogenic role in CRC by regulating PI3K/AKT signaling and is a potential biomarker for CRC diagnosis and prognostic monitoring.

## 1. Introduction

Colorectal cancer (CRC) is associated with the third highest morbidity and second highest mortality among all cancer types worldwide,^1^ seriously affecting patient quality of life. While progress in diagnosis and treatment in recent years has greatly improved the prognosis of CRC patients, prevention and disease management remain suboptimal. As the pathogenesis of CRC is still unclear, elucidating the molecular mechanisms driving CRC occurrence and development is of great significance for the discovery of early diagnostic markers and therapeutic targets.

Eukaryotic initiation factor 3a (eIF3a), located on human chromosome 10q26, is the largest and most complex subunit of eukaryotic translation initiation factor 3 (eIF3).^2^ Initial studies confirmed that eIF3a not only interacts with eIF3d, eIF3b, eIF3f, eIF3g, eIF3h, eIF3i, eIF3j, and eIF3k of the eIF3 family through its specific RNA domain,^3,4^ but also with cytokeratin 7, α-tubulin, nerve growth factor receptor tyrosine kinase TrKA, as well as other proteins and transcripts to regulate gene expression at the translational level.^5^ Through its various interactions, eIF3a affects multiple major processes, such as the cell cycle, differentiation, as well as DNA replication and repair.^6,7^ Haybaeck reported that eIF3a was upregulated in CRC tissue, and its expression was related to tumor stage.^8^ However, the molecular mechanisms through which eIF3a regulates CRC occurrence and development remain unknown.

In this study, we first analyzed the expression levels of eIF3a in patient peripheral blood and tumor tissue as well as from CRC cell line data in online databases (GEO and CCLE). We then determined the effects of eIF3a knockdown on cell proliferation, apoptosis, and EMT of CRC *in vitro* and *in vivo*. Finally, we conducted bioinformatics analysis to explore the underlying mechanism of eIF3a. Our findings indicated that silencing eIF3a inhibited CRC malignancy through a suppression of PI3K/AKT signaling.

## 2. Results

### 2.1. eIf3a expression is upregulated in CRC

**Figure 1.**
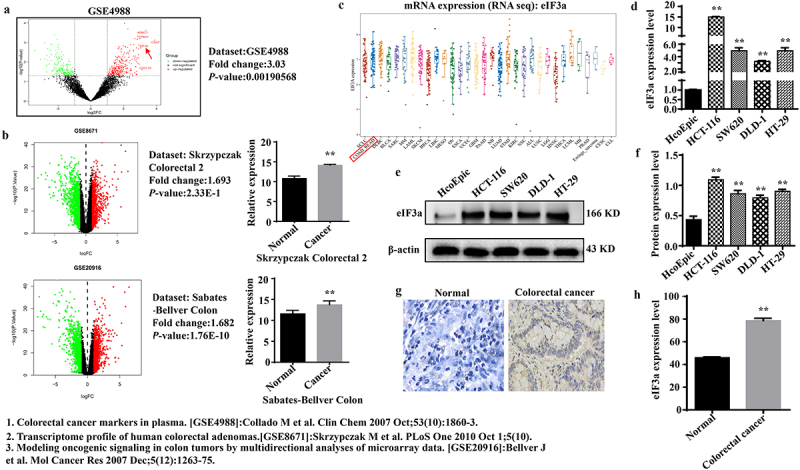
eIf3a was highly expressed in colorectal cancer (CRC). (a) eIF3a mRNA is highly expressed in the peripheral blood of CRC patients (GSE4988); (b) eIf3a mRNA was highly expressed in the tissues of CRC patients (GSE8671, GSE20916); (c) eIf3a mRNA expression in CRC lines (CCLE); (d) eIf3a mRNA expression in HCT116, SW620, HT29, and DLD-1 was higher than that in colonic epithelial cells (HcoEpic), ** *p* < .01 vs HcoEpic; (e,f) eIf3a protein expression was higher in CRC lines than in HcoEpic, ** *p* < .01 vs HcoEpic; (g,h) eIf3a was highly expressed in CRC patient tissue, ** *p* < .01 vs normal.

The eIF3a expression was examined in the peripheral blood and tumor tissues of CRC patients, as well as in CRC cell lines, utilizing publicly accessible datasets. eIF3a was highly expressed in the peripheral blood of CRC patients (*p <* 0.01, log FC = 3.03) (Figure 1a) as well as in patient tumor tissues (Figure 1b) and CRC cell lines (Figure 1c). Further, eIF3a mRNA (Figure 1d) and protein (Figure 1e,f) expression was significantly higher (*p* < .01) in CRC cell lines (HCT116, SW620, HT29, DLD-1) than in colonic epithelial cells (HcoEpic). IHC staining confirmed that eIF3a was significantly upregulated in patient CRC tissue than in paracancerous tissues (Figure 1g,h) (*p* < .01). Taken together, eIF3a was upregulated in CRC and thus may be involved in its occurrence and development.

### 2.2. Silencing eIf3a inhibits CRC cell proliferation

**Figure 2.**
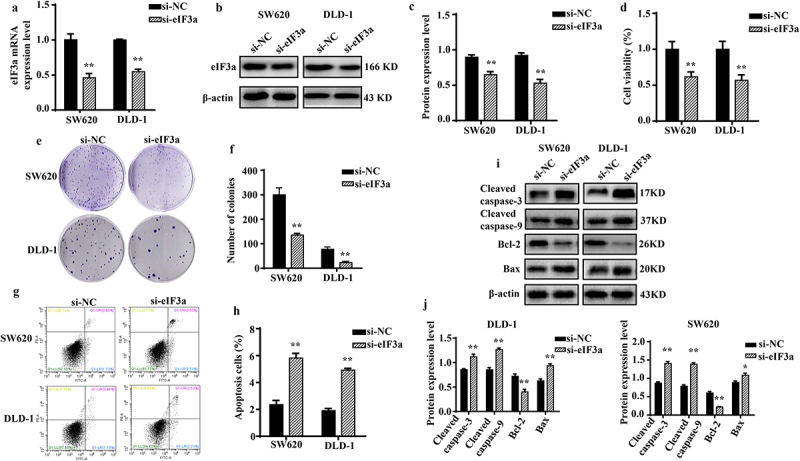
Silencing eIf3a inhibits colorectal cancer (CRC) cell proliferation. (a) eIF3a mRNA expression following si-eIf3a transfection of SW620 and DLD-1 cells was detected using real-time PCR; (b, c) eIf3a protein levels following si-eIf3a transfection were detected via western blotting; (d-f) Cell proliferation following si-eIf3a transfection was determined via MTT (D) and colony formation assay (e, f); (g, h) the apoptosis rate of cells following si-eIf3a transfection was detected using Annexin V-FITC/PI flow cytometry; (i, j) the effect of eIf3a silencing on apoptosis-related proteins was detected via western blot following si-eIf3a transfection. * *p* < .05, ** *p* < .01 vs si-NC group.

To investigate the biological function of eIF3a in CRC, endogenous eIF3a mRNA in SW620 and DLD-1 cells was silenced via transfection with si-eIF3a (Figure 2a), which led to a decrease in eIF3a protein levels (*p* < .01), as confirmed via western blotting (Figure 2b,c). Upon silencing eIF3a in both CRC lines, cell growth was significantly inhibited compared to the si-NC group (*p* < .01) (Figure 2d), with consistent results obtained in colony formation assay (*p* < .01) (Figure 2e,f). To further determine why silencing eIF3a inhibited cancer cell proliferation, flow cytometry and Hoechst -33,342 staining were performed to assess cell cycle progression and apoptosis. Compared with the si-NC group, cells in the si-eIF3a group were blocked at the G2 phase (*p* < .01) (Supple. Figure S1a,b), and the expression of cycle regulator Cyclin D1 was significantly reduced (*p* < .01) (Supple. Figure S1c,d). The apoptosis rate was significantly greater among silenced cells (*p* < .01) (Figure 2g,h), which exhibited typical apoptotic morphology (Supple. SFigure 1e), with increased expression of Cleaved caspase-3 and −9 (*p* < .01), pro-apoptotic protein Bax (*p* < .01), and decreased expression of the anti-apoptotic protein Bcl-2 (*p* < .01) (Figure 2i,j). Collectively, these findings indicate that silencing eIF3A can inhibit CRC proliferation by inducing apoptosis and concurrently blocking the cell cycle.

### 2.3. Silencing eIf3a inhibits the epithelial-to-mesenchymal transition (EMT) of CRC cells

**Figure 3.**
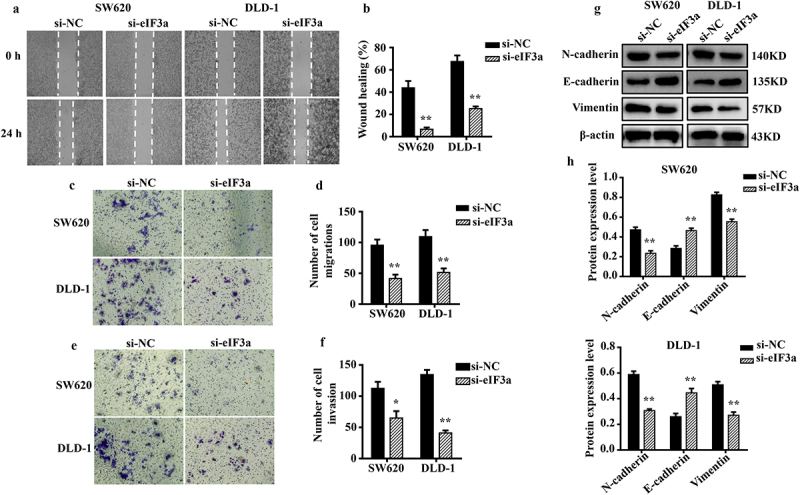
Silencing eIf3a inhibited the EMT of colorectal cancer (CRC) cells. (a, b) the motility of SW620 and DLD-1 cells following si-eIf3a transfection was determined via scratch wound assay; (c, d) the migratory ability of cells following si-eIf3a transfection was determined via Transwell assay; (e, f) the invasive ability of cells following si-eIf3a transfection was determined via Transwell (matrigel) assay; (g, h) EMT-related protein levels following si-eIf3a transfection of cells were detected via western blotting. * *p* < .05, ** *p* < .01 vs si-NC group.

In addition to having greater proliferative capacity than normal cells, tumor cells more frequently undergo EMT.^9^ We evaluated the motility, invasion, and migratory ability of SW620 and DLD-1 cells after silencing eIF3a. Cells in the si-eIF3a group exhibited reduced motility (*p* < .01) (Figure 3a,b), migration (*p* < .01) (Figure 3c,d), and invasion based on basement membrane crossing (*p* < .01) (Figure 3e,f) when compared to cells in the si-NC group. In addition, silencing eIF3a decreased the expression of mesenchymal markers N-cadherin and Vimentin in SW620 and DLD-1 cells (*p* < .01), while upregulating that of E-cadherin (*p* < .01) (Figure 3g,h). These findings suggest that suppressing eIF3a expression in CRC cells can effectively inhibit the EMT.

### 2.4. eIf3a overexpression promotes CRC proliferation and the EMT

**Figure 4.**
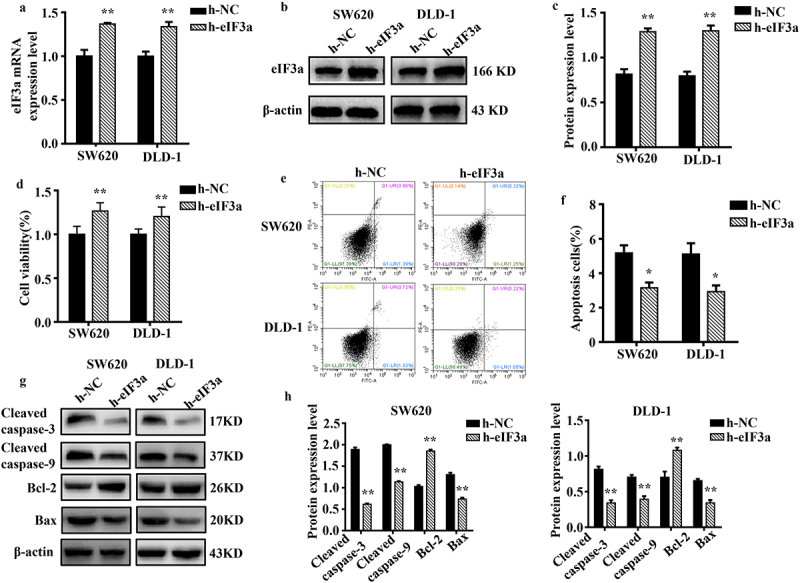
Overexpression of eIf3a inhibited apoptosis and promoted the proliferation of colorectal cancer (CRC) cells. (a) eIF3a mRNA expression levels in h-eIf3a-transfected SW620 and DLD-1 cells were detected using real-time PCR; (b, c) eIf3a protein levels following h-eIf3a transfection were detected via western blotting; (d) Cell proliferation following h-eIf3a transfection was detected using MTT assay; (e, f) the apoptosis rate of cells following h-eIf3a transfection was detected using Annexin V-FITC/PI flow cytometry; (g, h) the expression levels of apoptosis-related proteins following h-eIf3a transfection were detected via western blotting. * *p* < .05, ** *p* < .01 vs h-NC group.

**Figure 5.**
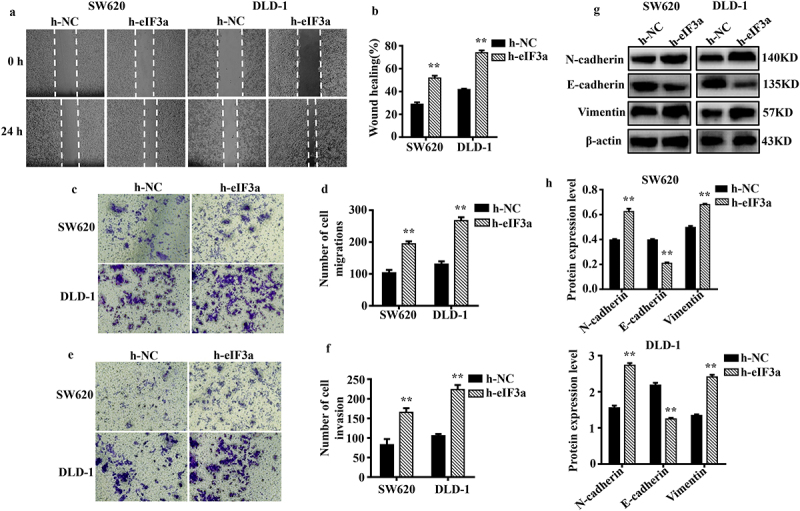
Overexpression of eIf3a promoted the EMT of colorectal cancer (CRC) cells. (a, b) the motility of SW620 and DLD-1 cells following transfection with h-eIf3a was determined via scratch wound assay; (c, d) the migratory ability of cells following transfection with h-eIf3a was determined via Transwell assay; (e, f) the invasive ability of cells following transfection with h-eIf3a was determined via Transwell (matrigel) assay; (g, h) EMT-related protein levels following h-eIf3a transfection were detected via western blotting. * *p* < .05, ** *p* < .01 vs h-NC group.

To further clarify its regulatory role in CRC cell proliferation and EMT, eIF3a was overexpressed in SW620 and DLD-1 cells. We evaluated changes in proliferation, cycle progression, apoptosis, migration, motility, and invasive ability. The mRNA and protein expression of eIF3a were significantly increased (*p* < .01) in both SW620 and DLD-1 cells upon eIF3a overexpression (Figure 4a–c). In parallel, cell proliferation was significantly enhanced (*p* < .01) (Figure 4d) (Supple. Figure S2a,b), with a shortened cell cycle duration (*p* < .05) (Supple. Figure S2,d), an increase in Cyclin D1 expression (*p* < .01) (Supple. Figure S2e,f), and a significantly lower apoptosis rate (*p* < .05) (Figure 4e,f). In addition, apoptotic morphology was not as pronounced (Supple. Figure S2g). Cleaved caspase-3, −9, and Bax were downregulated (*p* < .01), while Bcl-2 expression was increased (*p* < .01) (Figure 4g,h). Furthermore, cells in the h-eIF3a group exhibited greater motility (*p* < .01) (Figure 5a,b), migration (Figure 5c,d), and invasion (*p* < .01) (Figure 5e,f) when compared to those in the h-NC group. The expression of EMT-related markers N-cadherin and Vimentin was increased (*p* < .01), while E-cadherin was downregulated (*p* < .01) (Figure 5g,h). Through overexpression, eIF3a enhanced CRC cell proliferation and progression of the cell cycle, while also promoting EMT and inhibiting apoptosis. These findings provide further evidence that eIF3a contributes to the development of malignancy in CRC.

### 2.5. Silencing eIf3a inhibits CRC cell growth in vivo

**Figure 6.**
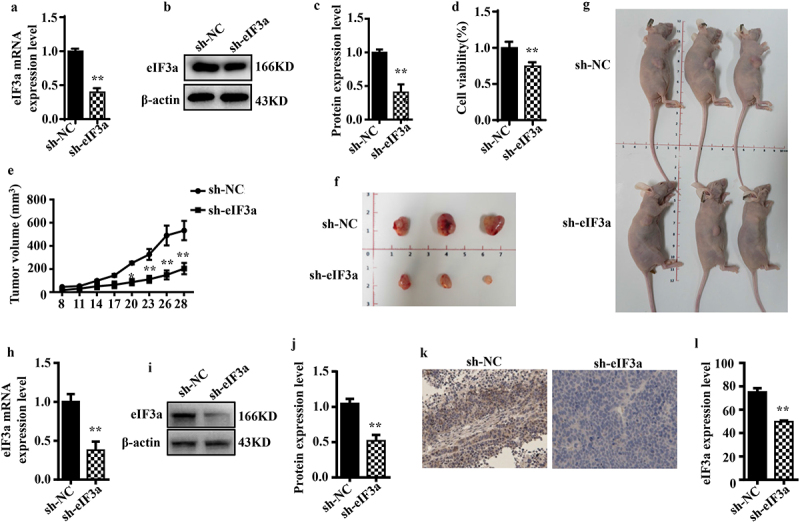
Silencing eIf3a inhibited the growth of colorectal cancer (CRC) *in vivo*. (a) eIF3a mRNA expression levels in SW620 cells with stably silenced eIf3a was detected using real-time PCR; (b, c) eIf3a expression levels in SW620 cells with stably silenced eIf3a were detected via western blotting; (d) the proliferation of SW620 cells with stably silenced eIf3a was detected via MTT assay; (e) the growth of eIf3a-silenced SW620 ×enograft tumors in nude mice; (f, g) the volume of tumors; (h) eIf3a mRNA expression levels in tumor tissues were detected via real-time PCR; (i, j) eIf3a protein levels in tumor tissues detected via western blotting; (k, l) eIf3a expression in tumor tissues determined via IHC staining. * *p* < .05, ** *p* < .01 vs sh-NC group.

To further investigate the effect of eIF3a on tumor growth, we established a SW620 cell line wherein eIF3a was stably silenced and then examined malignant behaviors. The results were consistent with those following siRNA-mediated silencing, with a reduction in mRNA and protein eIF3a expression (*p* < .01) (Figure 6a–c), reduced proliferation (*p* < .01) (Figure 6d), prolonged cell cycle duration (*p* < .01), increased apoptosis rate (*p* < .01), as well as suppressed migration and invasion ability (*p* < .05) (Supple. Figures S3 and S4). We then followed the growth of stably transduced cells in a nude mouse tumor xenograft model. Compared with the sh-NC cell group, sh-eIF3a mice exhibited significantly slower tumor growth (*p <* 0.01) (Figure 6e), resulting in a significantly smaller tumor volume (*p* < .01) (Figure 6f,g). In tumor tissues, the expression of eIF3a mRNA and protein was notably reduced in sh-eIF3a mice compared to those in the sh-NC group (*p* < .01) (Figure 6h–j). Furthermore, tumor eIF3a expression was lower in the sh-eIF3a group (Figure 6k,l), as confirmed by IHC staining (*p* < .01). These results suggested that silencing eIF3a can effectively inhibit CRC growth *in vivo*.

### 2.6. eIf3a promotes CRC malignant behaviors through the regulation of PI3K/AKT signaling

**Figure 7.**
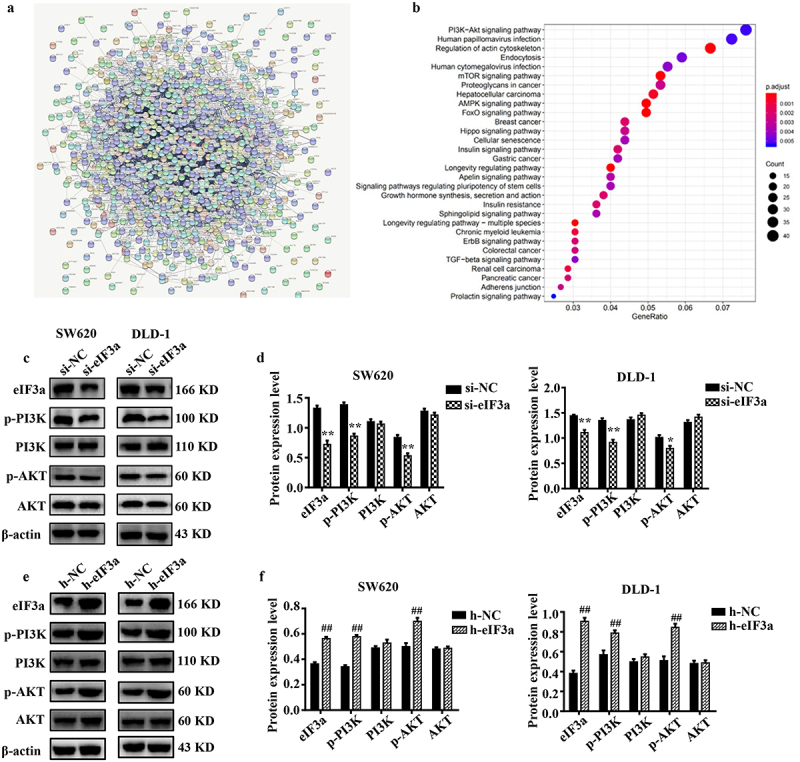
eIf3a affected the activation of PI3K/AKT signaling. (a) ENCORI enrichment analysis of eIf3a-interacting proteins; (b) KEGG pathway enrichment; (c, d) Effect of si-eIf3a in SW620 and DLD-1 cells on PI3K/AKT phosphorylation; (e, f) Effect of eIf3a overexpression in SW620 and DLD-1 cells on PI3K/AKT phosphorylation. * *p* < .05, ** *p* < .01 vs si-NC group, ^#^ *p* < .05, ^##^ *p* < .01 vs h-NC group.

To further elucidate the molecular mechanism through which eIF3a promotes the malignant behaviors of CRC cells, genes co-expressed with eIF3a were determined based on publicly available expression data (Figure 7a), and downstream signaling pathways were predicted through ENCORI enrichment analysis (Figure 7b). PI3K/AKT signaling was enriched with the highest number of related genes, and the enrichment was statistically significant. Therefore, eIF3a may play a role in promoting CRC development through the regulation of PI3K/AKT signaling.

We then validated the results of bioinformatics analysis, observing a significant decrease (*p* < .01) in p-PI3K and p-AKT levels in eIF3a-silenced SW620 and DLD-1 cells compared to cells of the si-NC group (Figure 7c,d). Meanwhile, there was a significant increase (*p* < .01) in p-PI3K and p-AKT levels in eIF3a-overexpressing cells relative to those of the h-NC group (Figure 7e,f). Consistent results were also obtained in SW620 cells where eIF3a was stably or transiently silenced (Supple. Figure S5). These results confirmed the involvement of PI3K/AKT signaling in eIF3a-mediated regulation.

**Figure 8.**
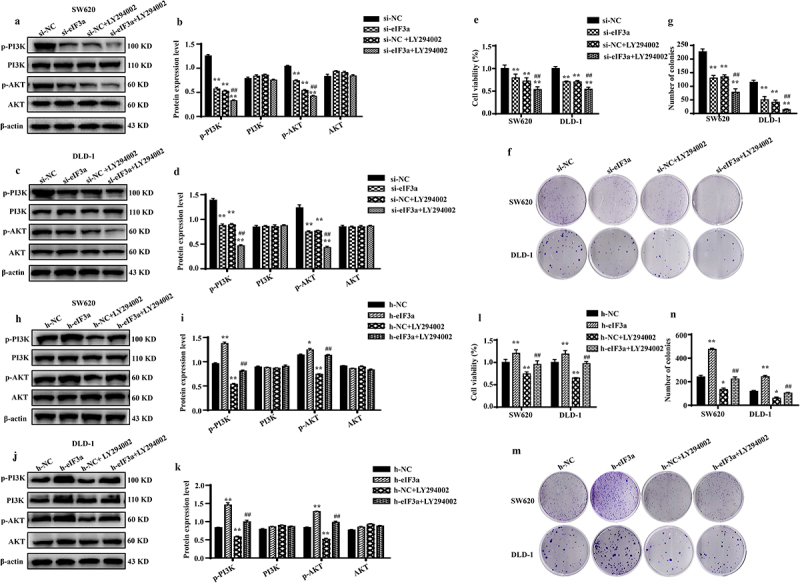
eIf3a activated PI3K/AKT signaling to promote colorectal cancer (CRC) cell proliferation. (a–d) Effect of si-eIf3a and PI3K/AKT-specific inhibitor LY294002 on eIf3a and PI3K/AKT signaling; (e-g) Effect of si-eIf3a and LY294002 on SW620 and DLD-1 cell proliferation; * *p* < .05, ** *p* < .01 vs NC group; ^#^ *p* < .05, ^##^ *p* < .01 vs si-NC+LY294002 group. (h-k) Effect of h-eIf3a and PI3K/AKT signaling pathway-specific inhibitor LY294002 on eIf3a and PI3K/AKT signaling; (l-n) Effect of h-eIf3a and LY294002 on SW620 and DLD-1 cell proliferation. * *p* < .05, ** *p* < .01 vs NC group; ^#^ *p* < .05, ^##^ *p* < .01 vs h-NC+LY294002 group.

To further verify the regulatory effect of eIF3a on the PI3K/AKT signaling pathway, eIF3a-silenced and eIF3a-overexpressing SW620 and DLD-1 cells were further treated with PI3K/AKT inhibitor LY294002. Both eIF3a silencing and LY294002 treatment significantly reduced the phosphorylation of the cellular PI3K/AKT signaling pathway compared to the si-NC group (*p* < .01). The inhibitory effect of eIF3a on PI3K/AKT phosphorylation was similar to LY294002 (*p* > .05), whereas LY294002 enhanced the impact of eIF3a on PI3K/AKT phosphorylation (*p* < .01) (Figure 8a–d), resulting in a more pronounced suppression of cell proliferation (*p* < .01) (Figure 8e–g). Similar results were obtained in SW620 cells stably transfected with eIF3a (Supple. Figure S6). Conversely, following LY294002 treatment in eIF3a-overexpressing cells, the level of PI3K/AKT phosphorylation significantly rose in the overexpression group (*p* < .01) but declined in the LY294002 group (*p* < .01) compared to the h-NC group. Furthermore, eIF3a overexpression reversed the inhibitory impact of LY294002 on PI3K/AKT phosphorylation (*p* < .01) (Figure 8h–k) and reversed LY294002‘s inhibitory effect on cell growth (*p* < .01) (Figure 8l–n). These findings suggest that the involvement of eIF3a in regulating the malignant biological behaviors of CRC is mediated via the PI3K/AKT signaling pathway.

## 3. Discussion

eIF3a (eIF3 p170) is the largest subunit of the eIF3 complex and has been suggested to regulate the translation of transcripts implicated in cell cycle progression and cell proliferation.^10^ During the past years, eIF3a has been recognized as a proto-oncogene.^11^ eIF3a is highly expressed in CRC,^12^ thyroid cancer,^13^ urinary bladder cancer,^14^ pancreatic ductal adenocarcinoma,^15^ hepatocellular carcinoma,^16^ ameloblastoma,^17^ cervix cancer,^18^ esophagus cancer,^19^ lung cancer,^2^ stomach cancer,^20^ and ovary cancer.^21^ Mei et al. reported that eIF3a was significantly upregulated in tumor tissues when compared with adjacent normal tissues, in addition to being correlated with tumor metastasis and overall survival.^12^ However, the specific mechanism of eIF3a implicated in CRC development and progression remains elusive.

In the present study, we observed that eIF3a was upregulated in peripheral blood and tumor tissues of CRC patients as well as in CRC cells lines, consistent with previous findings.^12^ We then systematically investigated the biological functions and associated molecular mechanism of eIF3a in CRC.

First, we analyzed the effects on proliferation, apoptosis, cell cycle progression, migration, and invasion of CRC cells. Silencing eIF3a effectively suppressed the malignant behaviors of CRC cells, while overexpression promoted these. Previous studies have shown that silencing eIF3a is effective in suppressing cell proliferation and EMT in thyroid,^13^ liver,^16^ and pancreatic cancer.^15^ The present results support a role for eIF3a as an oncogene in CRC. To further its regulatory role in CRC development and progression, we generated stably eIF3a-silenced CRC cells and established a nude mouse tumor xenograft model. Based on the observed suppression in cell growth, eIF3a was confirmed to play an oncogenic role in CRC.

Recent studies have shown that eIF3a may act alone or in complex with other eIF3 subunits necessary for translation initiation.^22^ In addition to regulating translation, an increasing number of studies have confirmed that eIF3a is implicated in cell cycle progression, cell differentiation, as well as DNA synthesis and repair.^6,7^ eIF3a can promote the migration of CRC by upregulating Cdc42 and RhoA expression at the translational level.^12^ Still, the specific molecular mechanisms through which eIF3a promoted CRC malignancy has never been investigated in depth.

To determine these, we employed bioinformatics analysis, which revealed PI3K/AKT signaling as the most significantly enriched in eIF3a co-expressed genes. The PI3K-AKT signaling pathway is among the commonly dysregulated signaling cascades in tumors, controlling cell growth, proliferation, differentiation, apoptosis, invasion, metastasis, and the EMT.^23^ Studies on other subunits of eIF3 have revealed that eIF3b, eIF3d, and eIF3c all promote tumor progression through PI3K/AKT signaling.^24–26^ Thus, PI3K/AKT may also be regulated downstream of eIF3a. We confirmed that both eIF3a-silenced and eIF3a-overexpressing CRC cells exhibit changes in PI3K/AKT activity, that is, activation was positively correlated with eIF3a expression. These findings confirmed the involvement of PI3K/AKT in eIF3a-mediated CRC malignancy. Next, eIF3a-silenced or eIF3a-overexpressing CRC cells were treated with a specific PI3K inhibitor (LY294002). LY294002 suppressed PI3K/AKT activation, as did eIF3a silencing. Further, LY294002 compromised the effect of eIF3a overexpression on PI3K-AKT activation. Both eIF3a silencing and LY294002 treatment suppressed cell proliferation, with LY294002 eliminating the proliferative effect of eIF3a overexpression in CRC cells.

In conclusion, the present study confirmed that eIF3a acts as an oncogene in CRC, as silencing it effectively inhibited various malignant behaviors, with PI3K/AKT signaling likely playing a key role in mediating the effects of eIF3a as an oncogene.

## 4. Materials and methods

### 4.1. Public data collection

We downloaded the GSE4988 dataset from GEO (https://www.ncbi.nlm.nih.gov/gds/) to obtain plasma sample mRNA expression data for CRC patients and healthy subjects. We also downloaded datasets (Sabates Bellver colon and Skrzypczak colorectal 2) from oncomine (www.oncomine.org) to obtain mRNA expression data for patient CRC tissues. Finally, we downloaded the dataset from CCLE (https://portals.broadinstitute.org/ccle/about) and constructed analysis using the R v4.0.3 software package ggplot2 (v3.3.3) to obtain eIF3a mRNA expression in CRC cell lines. Differential gene expression analysis was performed based on a *p* < .01 and a fold change of > 1.5.

### 4.2. Cell culture

Human CRC cells (SW620, DLD-1, HT-29, HCT-116) and human normal colon epithelial cells (HcoEpic) were cultured in Dulbecco’s Modified Eagle Medium with 4.5 g/L glucose (DMEM, Gibco BRL) containing 10% fetal bovine serum (FBS, Gibco BRL) and 1% antibiotic/antimycotic solution. Cells were maintained at 37°C in an atmosphere of 5% CO_2_.

### 4.3. Tissue sample collection

Paraffin-embedded sections for 30 pairs of primary CRC tissue and matched adjacent normal colorectal tissue were obtained from Shenzhen Nanshan District People’s Hospital. None of these patients had received preoperative chemotherapy or radiotherapy. This study was approved by the Human Body Research Ethics Committee of Shenzhen Nanshan District People’s Hospital.

### 4.4. Immunohistochemistry (IHC)

IHC was used to detect eIF3a expression in paired tissues. The antibody of eIF3a was purchased from Abcam (Cambridge, UK) and the secondary antibody (rabbit-IgG) was obtained from Cell Signaling Technology (Beverly, MA, USA).

### 4.5. RNA extraction and quantitative real-time PCR

Total RNA was extracted from cells using Trizol reagent (Sangon Biotech, CHN) according to the manufacturer’s instructions. Real-time PCR reactions were performed using SYBR Premix Ex Taq™ II (Takara, JPN) on the BIO-RAD CFX96 Real-Time PCR System. The following primers were used: β-actin forward 5’-AGGATGCAGAAGGAGATCAC-3’ and reverse 5’-TGTAACGCAACTAAGTCA

TAG-3’, eIF3a forward 5’-TGGAACTTTGCGTGGATCTTCG-3’ and reverse 5’-AGA

CCATCTGCTGAGATTCTTC −3’.

### 4.6. Western blot

Western blot was carried out as previously described.^27^ The antibody against eIF3a was purchased from Abcam (Cambridge, UK), and the β-actin antibody was purchased from Santa Cruz. Antibodies against Phospho-PI3 Kinase Class III (Ser249), PI3K Kinase P110e (C73F8), Phospho-AKT (Ser473) (D9E), AKT (Pan) (C67E7), Cleaved caspase-9, Cleaved caspase-3, Bcl-2, Bax, E-cadherin (4A2), N-cadherin, and cleaved were purchased from Cell Signaling Technology (Beverly, MA, USA).

### 4.7. siRNA synthesis, plasmid construction and cell transfection

Purified, double-stranded siRNA against human eIF3a was purchased from Gene Pharma (Shanghai, China). Sequences of the sense strand and antisense strand are 5’-GCGAUCAUCCUGGCGUAAUTT-3’ and 5’-AUUACGCCAGGAUGAUCGCTT −3’ respectively. sh-eIF3a plasmid encoding shRNA against human eIF3a was designed on the basis of above mentioned eIF3a siRNA and constructed by Shenzhen Ritzcon Biological Technology (Shenzhen, China). h-eIF3a plasmid encoding human eIF3a open reading frame (ORF), for the purpose of overexpressing eIF3a in mammalian cells, was purchased from VectorBuilder (Guangzhou, China).

The eIF3a siRNA and above-mentioned plasmids were transfected into CRC cells with Xfect RNA Transfection Reagent (Takara). eIF3a mRNA and protein levels in CRC cells were analyzed via real-time PCR and western blot to determine transfection efficiency.

### 4.8. Stable cell establishment and characterization

Plasmid sh-eIF3a was transfected into CRC cells with Xfect RNA Transfection Reagent (Takara). On the next day, the cells were sub-cultured and treated with 5 μg/mL puromycin for 7 days. Then the medium was changed to 1 μg/ml puromycin supplemented and the cells were further culture for 7 days. Afterwards single cell colony was picked, cultured, and characterized for eIF3a expression. Cells with eIF3a expression suppressed were selected as stable cells.

### 4.9. Cell viability measurement

Transfected cells (4 × 10^3^ cells/well) were inoculated on 96-well plates and cultured for 48 h, whereafter 10% CCK8 (Dojindo, Rockville, USA) solution was added to each well and incubated for 2 h. A microplate autoreader (Bio-RWad, Hercules, CA, USA) was used to measure absorbance at a wavelength of 570 nm.

### 4.10. Colony formation assay

The transfected cells were seeded on 6-well plates (200 cells/well) and cultured for 2 weeks. The colonies were fixed with 4% paraformaldehyde (Sigma) for 30 min, stained with 0.01% crystal violet for 10 min, and counted under an inverted microscope.

### 4.11. Hoechst -33,342 staining

SW620 and DLD-1 cells (5 × 10^5^ cells/well) were inoculated into 6-well plates. After 48 h of transfection with an eIF3a expression plasmid or siRNA, cells were stained with Hoechst -33,342 DNA-binding dye (Beyotime, Guangzhou, China) (10 mg/L), and imaged under a fluorescence microscope.

### 4.12. Apoptosis and cell cycle assay

After 48 h of cell transfection, apoptosis and cell cycle were evaluated via flow cytometry (BD Accuri C6，Franklin Lake) using the Annexin V-FITC Apoptosis Detection Kit (Beyotime Biotechnology, Haimen, China), PI staining, as well as the Cell Cycle and Apoptosis Analysis Kit (Beyotime, HaiMen, China) as per manufacturer instructions. Results were then analyzed using FlowJo.

### 4.13. Scratch wound assay

The scratch wound assay was conducted as previously described. Cell motility was assessed using scratch wound assay. SW620 and DLD-1 cells (8 × 10^5^ cells/well) were inoculated into 6-well plates. After 24 h of transfection with the eIF3a-expressing plasmid or siRNA, cells were imaged via microscopy at 0 and 24 h. ImageJ was used to calculate motility.

### 4.14. Cell invasion and migration assay

Transwell chambers were used to assess CRC cell migration and invasion capacity. In brief, transfected cells were trypsinized and resuspended with culture medium lacking FBS. Cells (5 × 10^4^ cells/250 μL/well) were added to the upper chamber of inserts with or without Matrix gel. Meanwhile, 750 µL medium containing 20% FBS was added to the lower chamber. After incubation at a constant temperature in an incubator at 5% CO_2_ and 37°C for 48 h, cells were fixed with 4% paraformaldehyde for 15 min, followed by staining with crystal violet and imaging. Results were analyzed in ImageJ.

### 4.15. Xenograft mouse model

Female BALB/c nude mice (5–6 weeks old) were purchased from the GuangDong GemPhamatech Co., Ltd. Mouse experiments were approved by the Animal Experimental Ethics Committee of GDMU and followed Guangdong Medical University guidelines. 2 × 10^6^ stable eIF3a knockout and control SW620 cells suspended in 100 μL DMEM were subcutaneously injected into the right armpit of each mouse. Tumor size was measured every 3 days, and primary tumors were surgically removed for IHC, western blotting, and real-time PCR.

### 4.16. Kyoto Encyclopedia of Genes and Genomes (KEGG) pathway enrichment analysis

Using the online database ENCORI (https://starbase.sysu.edu.cn/), we obtained genes co-expressed with eIF3a. STRING (https://string-db.org/) was conducted to construct a protein-protein interaction (PPI) network, whereafter downstream signaling pathways were predicted via KEGG pathway enrichment analysis.

### 4.17. Statistical analysis

Data were analyzed via SPSS 19.0 and are expressed as the mean±SD. Comparisons were performed using an unpaired Student’s test, and differences between groups were analyzed via one-way ANOVA. *p* < .05 was considered to indicate statistical significance. GraphPad Prism 6.0 was used for to generate histograms.

## Supplementary Material

Supplemental Material

## Data Availability

The data used to support the findings of this study are available from the corresponding author upon request.

## Abbreviations

CRC: colorectal cancer
eIF3a: eukaryotic initiation factor 3a
IHC: Immunohistochemistry
KEGG: Kyoto Encyclopedia of Genes and Genomes
DAB: 3,3-diaminobenzidine
PPI: protein-protein interaction
