# Supplementary material for: eIf3a mediates malignant biological behaviors in colorectal cancer through the PI3K/AKT signaling pathway
Source: Cancer Biol Ther. 2024 May 23;25(1):2355703. doi: 10.1080/15384047.2024.2355703 (PMC11123456; doi:10.1080/15384047.2024.2355703)
Supplement: Supplemental Material [file KCBT_A_2355703_SM5807.zip › supplementary_files/Supplementary Figures legends.docx]

Supplementary Figure 1. Silencing eIF3a inhibits colorectal cancer (CRC) cell proliferation. (A, B) Cell cycle changes following si-eIF3a transfection of SW620 and DLD-1 cells were detected using flow cytometry; (C, D) Cyclin D1 expression following si-eIF3a transfection was detected via western blot; (E) Cellular apoptotic morphology following si-eIF3a transfection was assessed via Hoechst-33342 staining (red arrows). * *p* < 0.05, ** *p* < 0.01 vs si-NC group.

Supplementary Figure 2. Overexpression of eIF3a promoted the malignant behaviors of colorectal cancer (CRC) cells. (A, B) Cell proliferation following h-eIF3a transfection of SW620 and DLD-1 cells was detected using colony formation assay; (C, D) Cell cycle changes following h-eIF3a transfection were determined using flow cytometry; (E, F) Cyclin D1 expression levels following h-eIF3a transfection were detected via western blotting; (G) Cellular apoptotic morphology following h-eIF3a transfection was observed using Hoechst-33342 staining (red arrows). * *p* < 0.05, ** *p* < 0.01 vs h-NC group.

Supplementary Figure 3. Proliferation and apoptosis of SW620 cells with stably silenced eIF3a. (A, B) Cell proliferation was detected via colony formation assay; (C) Cellular apoptotic morphology was observed using Hoechst-33342 staining (red arrow); (D, E) The apoptosis rate of cells was determined using Annexin V-FITC/PI flow cytometry; (F, G) The levels of apoptosis-related proteins were determined via western blotting; (H, I) Cell cycle changes were determined via flow cytometry; (J, K) Cyclin D1 expression levels were detected using western blotting. * *p* < 0.05, ** *p* < 0.01 vs sh-NC group.

Supplementary Figure 4. EMT of SW620 cells with stably silenced eIF3a. (A, B) Cell motility was detected using scratch wound assay; (C, D) Cell migratory ability was determined via Transwell assay; (E, F) Cell invasive ability was determined via Transwell (matrigel) assay; (G, H) Levels of EMT-related proteins were detected via western blotting. * *p* < 0.05, ** *p* < 0.01 vs sh-NC group.

Supplementary Figure 5. Phosphorylation of PI3K/AKT in SW620 cells with stably silenced eIF3a. * *p* < 0.05, ** *p* < 0.01 vs sh-NC group.

Supplementary Figure 6. eIF3a-mediated activation of PI3K/AKT signaling promoted the malignant behaviors of colorectal cancer (CRC) cells. (A, B) Effect of PI3K/AKT signaling pathway-specific inhibitor LY294002 on PI3K/AKT phosphorylation in SW620 cells with stably silenced eIF3a; (C-E) Effect of PI3K/AKT signaling pathway-specific inhibitor LY294002 on the proliferation of SW620 cells with stably silenced eIF3a. * *p* < 0.05, ** *p* < 0.01 vs sh-NC group, ^#^ *p* < 0.05, ^##^ *p* < 0.01 vs sh-NC+LY294002 group.
