# Supplementary figures and images for: eIf3a mediates malignant biological behaviors in colorectal cancer through the PI3K/AKT signaling pathway
Source: Cancer Biol Ther. 2024 May 23;25(1):2355703. doi: 10.1080/15384047.2024.2355703 (PMC11123456; doi:10.1080/15384047.2024.2355703)

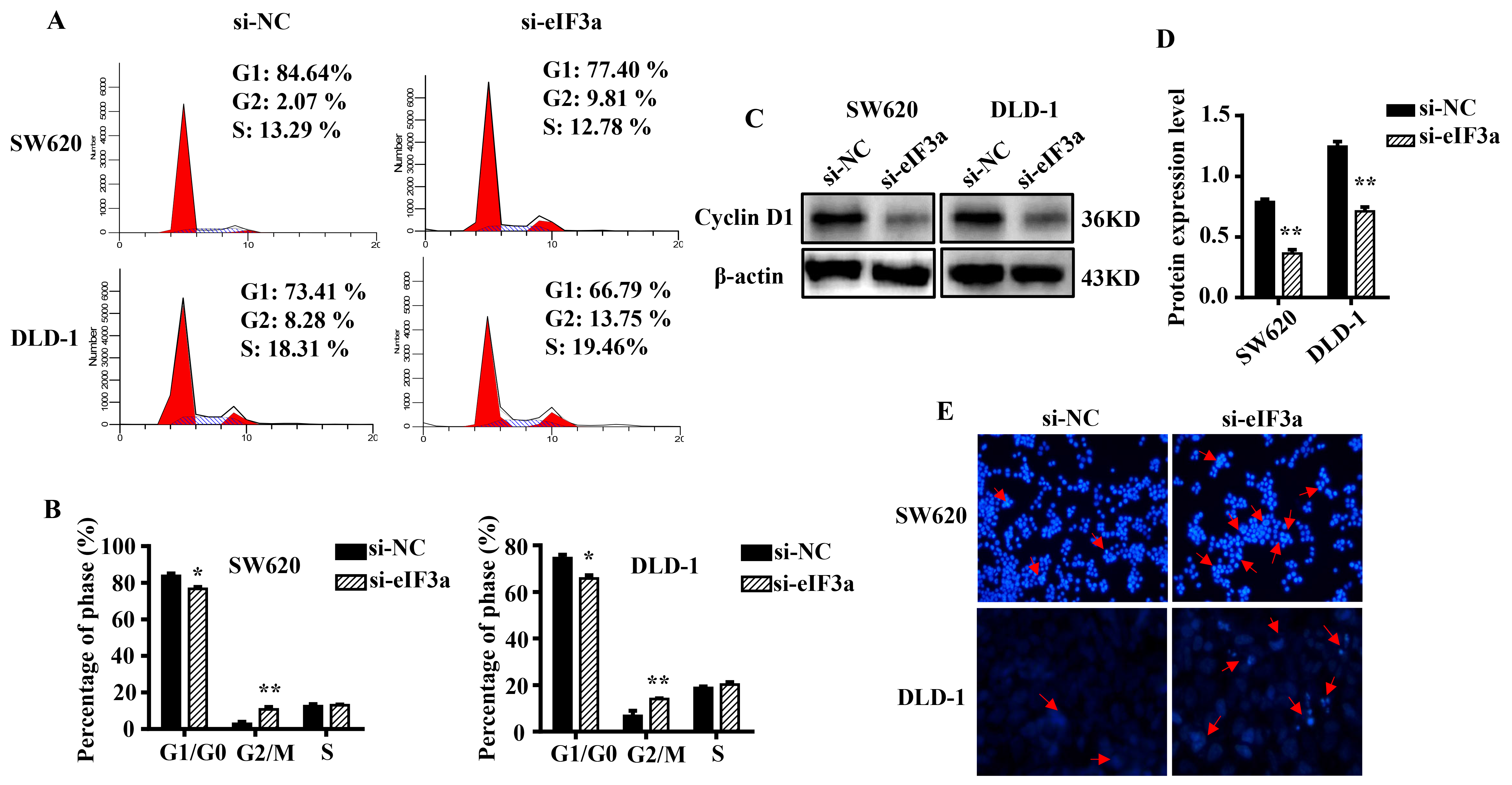

Supplement: Supplemental Material [file KCBT_A_2355703_SM5807.zip › supplementary_files/supplementary figure 1.tif]

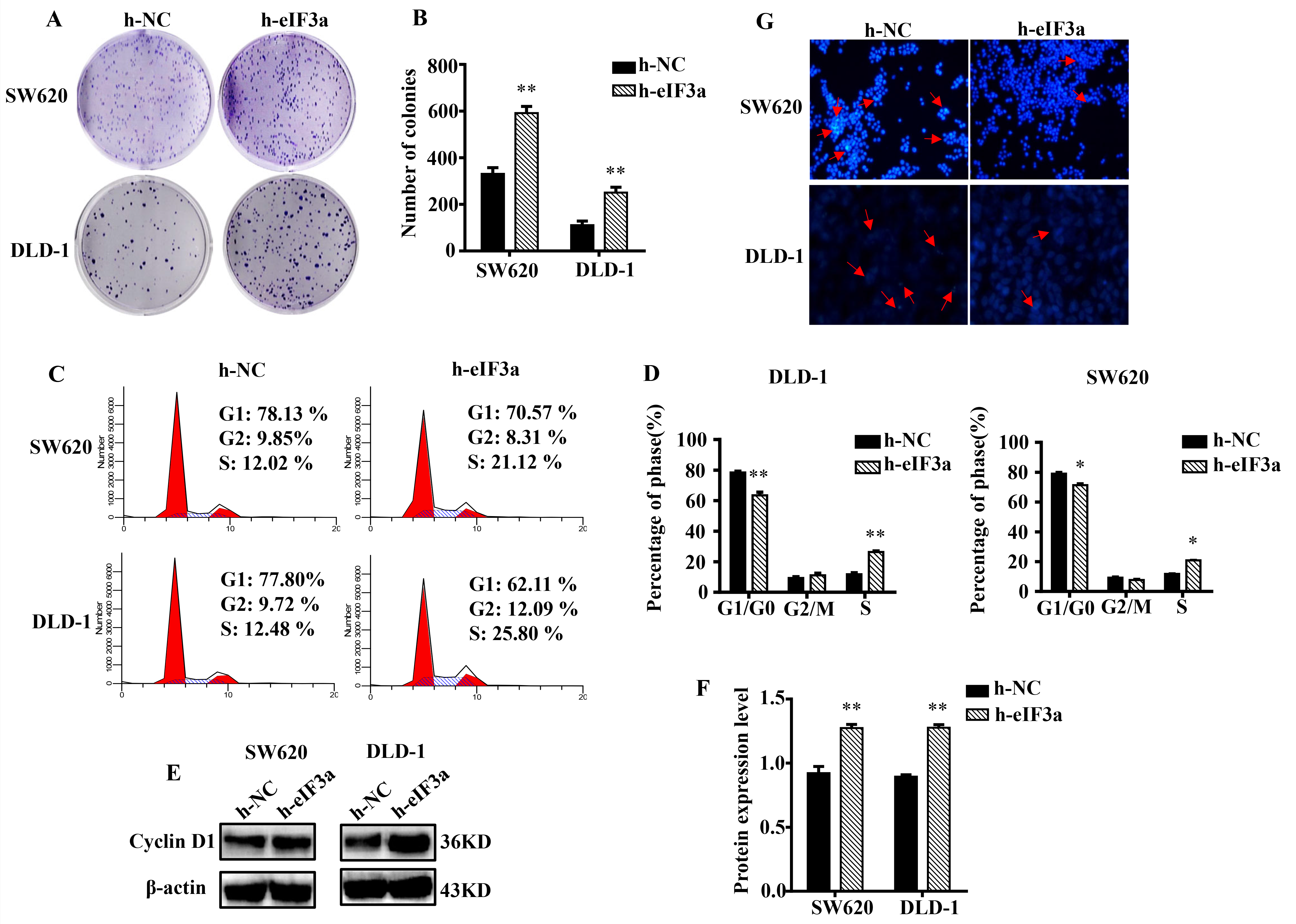

Supplement: Supplemental Material [file KCBT_A_2355703_SM5807.zip › supplementary_files/supplementary figure 2.tif]

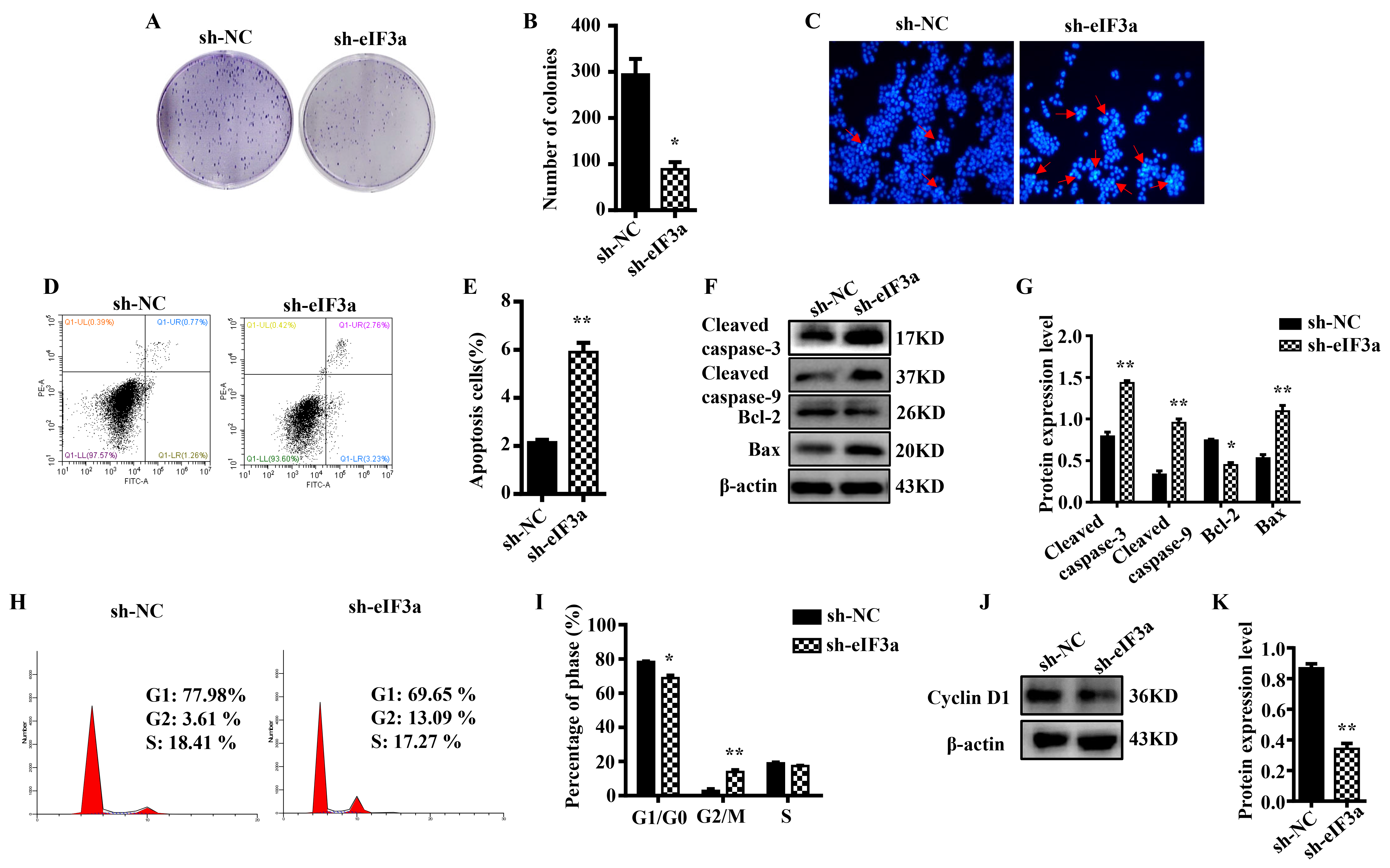

Supplement: Supplemental Material [file KCBT_A_2355703_SM5807.zip › supplementary_files/supplementary figure 3.tif]

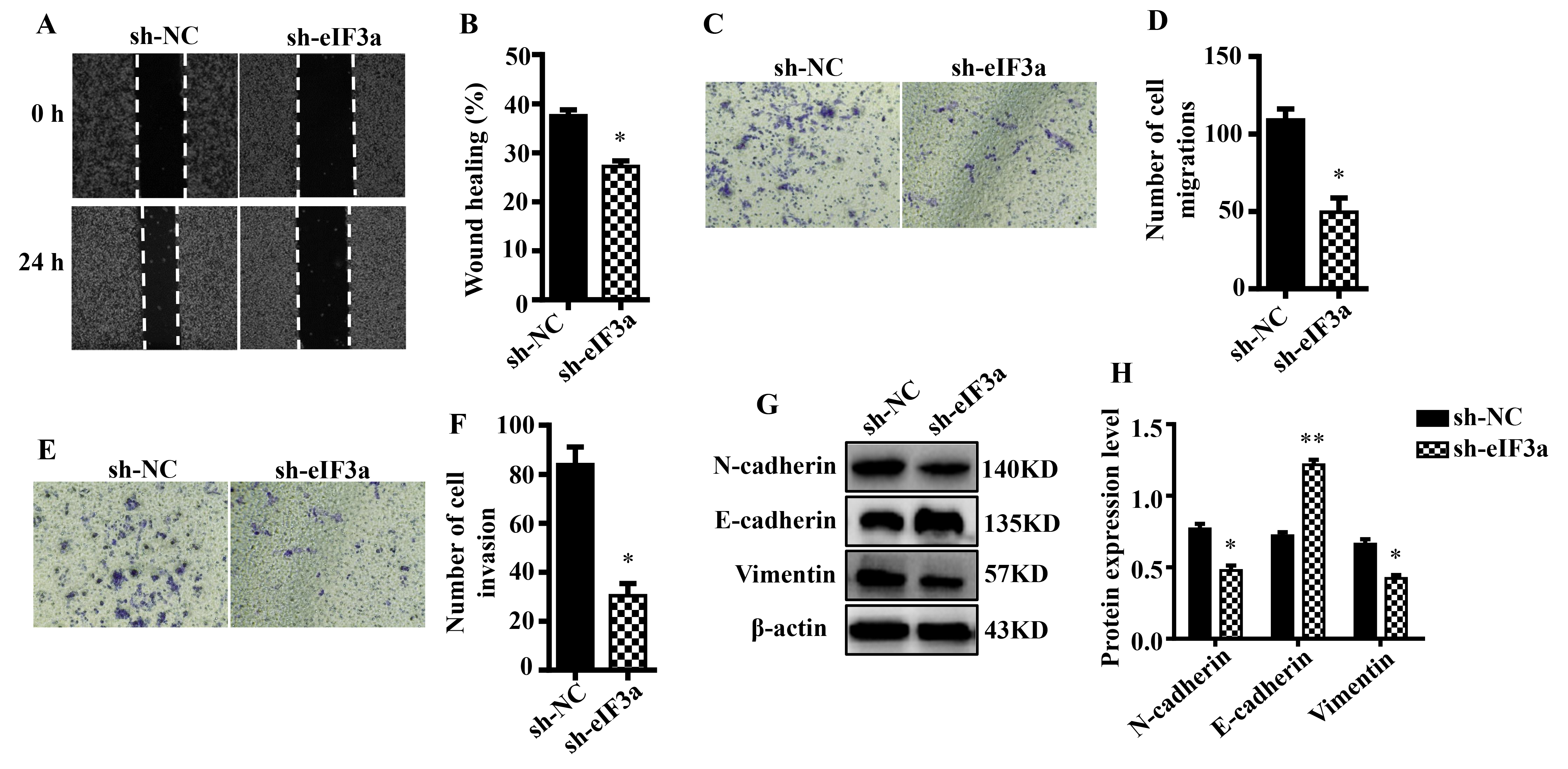

Supplement: Supplemental Material [file KCBT_A_2355703_SM5807.zip › supplementary_files/supplementary figure 4.tif]

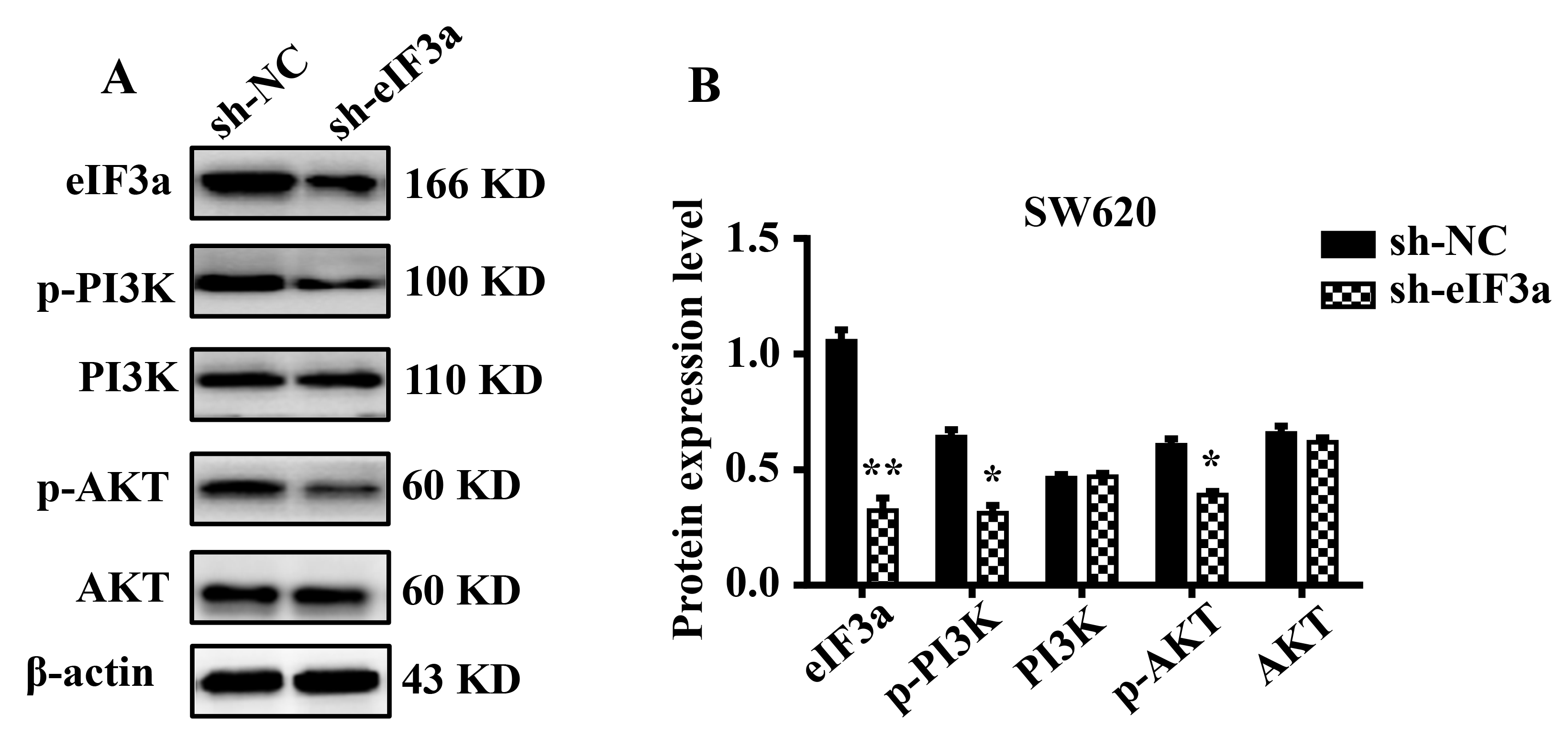

Supplement: Supplemental Material [file KCBT_A_2355703_SM5807.zip › supplementary_files/supplementary figure 5.tif]

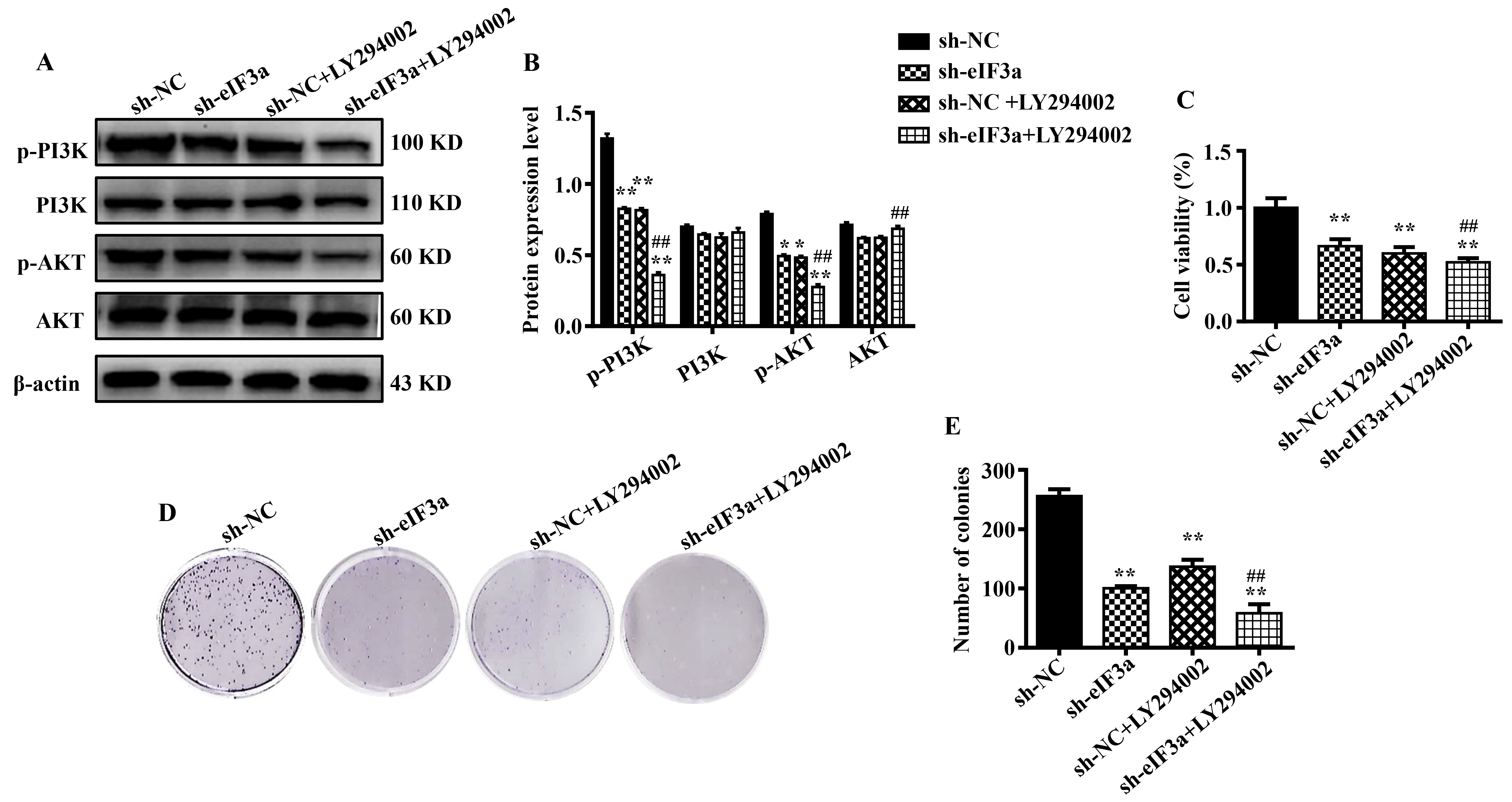

Supplement: Supplemental Material [file KCBT_A_2355703_SM5807.zip › supplementary_files/supplementary figure 6.tif]
